# Leaf and canopy reflectance spectrometry applied to the estimation of angular leaf spot disease severity of common bean crops

**DOI:** 10.1371/journal.pone.0196072

**Published:** 2018-04-26

**Authors:** Víctor Martínez-Martínez, Jaime Gomez-Gil, Marley L. Machado, Francisco A. C. Pinto

**Affiliations:** 1 Department of Signal Theory, Communications and Telematics Engineering, University of Valladolid, Valladolid, Spain; 2 Departamento de Pesquisa, Empresa de Pesquisa Agropecuária de Minas Gerais, Belo Horizonte, Minas Gerais, Brazil; 3 Departamento de Engenharia Agrícola, Universidade Federal de Viçosa, Viçosa, Minas Gerais, Brazil; University of the West of England, UNITED KINGDOM

## Abstract

This study is aimed at (i) estimating the angular leaf spot (ALS) disease severity in common beans crops in Brazil, caused by the fungus *Pseudocercospora griseola*, employing leaf and canopy spectral reflectance data, (ii) evaluating the informative spectral regions in the detection, and (iii) comparing the estimation accuracy when the reflectance or the first derivative reflectance (FDR) is employed. Three data sets of useful spectral reflectance measurements in the 440 to 850 nm range were employed; measurements were taken over the leaves and canopy of bean crops with different levels of disease. A system based in Principal Component Analysis (PCA) and Artificial Neural Networks (ANN) was developed to estimate the disease severity from leaf and canopy hyperspectral reflectance spectra. Levels of disease to be taken as true reference were determined from the proportion of the total leaf surface covered by necrotic lesions on RGB images. When estimating ALS disease severity in bean crops by using hyperspectral reflectance spectrometry, this study suggests that (i) successful estimations with coefficients of determination up to 0.87 can be achieved if the spectra is acquired by the spectroradiometer in contact with the leaves, (ii) unsuccessful estimations are obtained when the spectra are acquired by the spectroradiometer from one or more meters above the crop, (iii) the red to near-infrared spectral region (630–850 nm) offers the same precision in the estimation as the blue to near-infrared spectral region (440–850), and (iv) neither significant improvements nor significant detriments are achieved when the input data to the estimation processing system are the FDR spectra, instead of the reflectance spectra.

## 1. Introduction

*Phaseolus vulgaris*, the common bean, is a legume crop consumed worldwide. In 2012, 24 million tons of dry common beans and 21 million tons of green beans were produced, ranking as the 11^th^ and 78^th^ most produced crops worldwide, respectively [1, 2]. Beans are an excellent source of protein, carbohydrates, dietary fiber, vitamins, minerals, and phytochemicals [3]. Consumption of beans has health benefits because it can reduce cholesterol levels [4], diabetes [5], and the risk of prostate cancer [6] and mammary cancer [7]. Some studies suggest that diets rich in legumes, such as beans, are sometimes associated with longevity [8].

Several bacterial, fungal, and viral diseases attack aerial and underground parts of common beans [9–11]. Among fungal diseases, the angular leaf spot (ALS), caused by the fungus *Pseudocercospora griseola*, is responsible for important yield losses worldwide [12–14]. An early detection of ALS disease in a crop allows for effective disease management.

When light strikes a leaf, part of the light is reflected towards the observer. The amount energy reflected at each light frequency is named reflectance spectrum, sometimes abbreviated by spectra or by reflectance. Reflectance depends on leaf surface properties and internal structure, as well as by the concentration and distribution of biochemical components. Leaf and canopy reflectance can be used to diagnose plant status. In the visible spectrum, (VIS, between 400 and 700 nm) reflectance depends mainly on the presence of photosynthetic pigments such as chlorophyll. In the near infrared domain (NIR, between 700 and 13000 nm), where there are no strong absorption features, the magnitude of reflectance is governed by structural discontinuities encountered in the leaf. The shortwave infrared region (SWIR, between 1300 nm and 3000 nm), presents variable reflectance values mainly linked to the absorption characteristics of water and other compounds [15]. Diseased plants usually exhibit discrete lesions on leaves, corresponding to necrotic or chlorotic regions, with greater reflectance values in the visible range [16]. A sharp transition from low to high reflectance usually occurs in the wavelengths between the visible and the NIR regions (around 700 nm), and this transition usually shifts to shorter wavelengths in diseased crops [17]. The first derivative reflectance allows to easily compute the wavelength were this transition occurs. Characteristics extraction of the reflectance spectrum, and classification or regression techniques, can be used to diagnose the plant status.

Multispectral reflectance data, characterized by having spectral regions more than 40 nm wide, usually measure energy in between 2 and 10 separated regions. Spectral Vegetation Indices (SVIs) can be computed from combinations of some region values from the multispectral reflectance data. Employing multispectral reflectance data, and in some cases computing indices, to estimate disease in crops is common in the literature. For example, Dammer *et al*. [18] computed the NDVI index using the red and the infra-red regions to estimate head blight on wheat crops, Cui *et al*. [19] detected rust disease in soybean crops employing different vegetation indices, and Xiao and McPherson [20] determined the health of 215 tree species by means of the NDVI index.

Hyperspectral reflectance data, characterized by spectral regions less than 10 nm wide, are typically composed of 200 or more regions. It allows the in-depth examination of crop features, this not being possible with the relative coarse bandwidths acquired by multispectral spectrometers. Using hyperspectral reflectance data for disease estimation in crops is common in the scientific literature. For example, Zhang *et al*. [21] detected late blight disease in tomato crops caused by the fungal pathogen *Phytophthora infestans*, Muhammed *et al*. [22] quantified tan spot disease severity in wheat crops infected by the fungal pathogen *Pyrenophora tritici-repentis*, Wu *et al*. [23] detected *Botrytis cinerea* fungus infestation in eggplant crops, Liu *et al*. [24] classified in levels the severity of rice glume blight disease caused by the fungus *Microsphaeropsis glumarum*, and the severity of rice false smut disease caused by the fungus *Ustilaginoidea virens*, performing both classifications in rice crops, Prabhakar *et al*. [25] estimated yellow mosaic disease caused by *Mungbean yellow mosaic virus* in black bean, and Pietrzykowski *et al*. [26] detected *Mycosphaerella* leaf disease caused by several fungus in *Eucalyptus globulus* foliage, with an index computed from the reflectance at 550 nm and 678 nm.

Hyperspectral reflectance spectra contain high correlated information in a large number of wavelengths. Several methods have been employed to reduce the length of the reflectance vector using this correlation, such as the computation of SVIs [20, 27, 28], Principal Component Analysis (PCA) [24, 29, 30], Principal Component Regression (PCR) [31], and Partial Least Squares (PLS) regression [23, 28, 32]. Once that correlation is reduced, decision trees [33, 34], Artificial Neural Networks (ANNs) [24, 35, 36], Support Vector Machines (SVMs) [37–39], and clustering [33, 40, 41], among other techniques, are usually employed to estimate crop features in remote sensing.

This study aims to (i) estimate the ALS disease severity in common beans crops from leaf and canopy spectral reflectance data, (ii) evaluate the informative spectral regions in the detection of the disease, and (iii) compare the estimation accuracy when the reflectance data is employed to the one obtained when the first derivative reflectance (FDR) data is employed instead.

## 2. Material and methods

This section presents the study site where the experiments were performed, and the materials and methods employed to acquire the data and to process these data to estimate the disease severity.

### 2.1. Study site

Three experiments, named UFV1, UFV2, and FEVP3, were conducted in 2011. UFV1 and UFV2 were performed in the Diogo Alves de Melo experimental area of the Universidade Federal de Viçosa, in Viçosa, MG, Brazil (20° 45’ S, 42° 52’ W, altitude 648 m), and they consisted of 40 and 32 different plots, respectively. The FEVP3 site was in Vale do Piranga experimental area in Oratorios, MG, Brazil (20° 24’ S, 42° 48’ W, altitude 450 m), and it consisted of 40 different plots.

The soil was prepared with a disc harrow. The crops were seeded on April 8, July 29, and July 26 for UFV1, UFV2, and FEVP3, respectively. The variety of common beans employed was Ouro Vermelho, which is susceptible to ALS. The seed density was 10 seeds per meter along a 5.5-meter row, with five rows per plot and 0.5 meters between each row. Common insecticides and 8-28-16 fertilizer were applied while the crop was growing. Weeds were manually removed.

Bean straw from others nearby fields, affected by the ALS, was evenly spread in the plots eight days after the sprouting of the crop. The study was developed as a completely randomized design involving four treatments with 10, 8, and 10 replications for UFV1, UFV2, and FEVP3 experiments, respectively. The treatments considered four different doses of fungicide in order to achieve different ALS severities. The AMISTAR^®^ fungicide, whose active ingredient is azoxystrobin (23.1% w/w), was applied in doses of 0, 50, 100, and 150 g/ha of commercial product on each part of the plot. The fungicide was applied three times during the crop development using a back sprayer, which was previously calibrated to each fungicide dose.

### 2.2. Data acquisition

Three types of data were acquired: leaf reflectance spectra, canopy reflectance spectra, and leaf RGB images.

**Fig 1:**
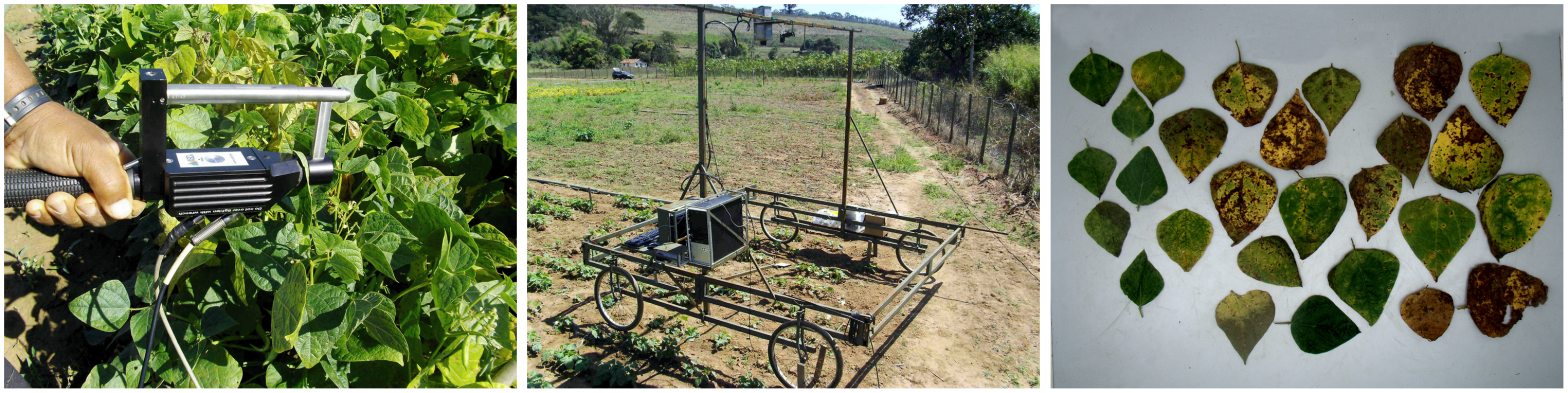
Representative images of the data acquisition. (a) spectroradiometer and leaf clip accessory employed to acquire leaf reflectance spectrum, (b) spectroradiometer and platform employed to acquire canopy reflectance, and (c) RGB image of several leaves analyzed.

Leaf reflectance spectral data in the 325–1075 nm spectral region with a resolution of 1 nm was acquired employing an ASD Field Spec HandHeld-2 spectroradiometer, manufactured by the Analytical Spectral Devices company, from Boulder, Colorado, USA. A leaf clip accessory, also from the same company, was connected to the spectroradiometer (Fig 1a). Calibrations were done using a spectralon white panel, following the guidelines of the spectroradiometer user manual. All the data was collected between 10:00 and 14:00 Brasilia Time (GMT-3). The acquisition software employed was RS3, which was provided by the manufacturer of the spectroradiometer. The data was exported by the ViewSpectro Pro software, which was also provided by the manufacturer of the spectroradiometer. Each leaf reflectance spectrum of the crop was considered as the mean of the reflectance spectra obtained with 27 leaves taken from nine different plants randomly chosen for each plot. From each plant, it was taken a leaf of the upper third, one of the middle third, and other of the lower third, measuring always the central part of each leaf. The reflectance readings of these leaves were made immediately after they were taken. Canopy reflectance spectral data was acquired by employing: the same spectroradiometer without the leaf clip accessory, a four-wheel metallic platform, and a four-meter-long optical fiber cable. This made it possible to focus the spectroradiometer on the canopy, from three meters above the ground (Fig 1b).

Leaf RGB images were acquired employing an ordinary digital camera, an 0.8×0.8×0.8 m wooden box with an opening at the top for camera positioning, and six fluorescent lamps illuminating the inside of the box. The 27 leaves of each plot collected to spectral readings were placed at the bottom of the box, and photos were taken from the opening at the top (Fig 1c). The effective resolution was 3.78 pixels/mm.

Table 1 presents the data collection times for canopy reflectance, leaf reflectance and leaf images on different crop growth stage, characterized as days after plant emergence. All these data is supplied as a Repository File, which is explained in S1 Text.

**Table 1:** Data collection times for leaf reflectance (LR), canopy reflectance (CR) and leaf image (LI) on different days after plant emergence (DAE) for each experiment.

UFV1				UFV2				FEVP3			
DAE	LR	CR	LI	DAE	LR	CR	LI	DAE	LR	CR	LI
33	x	x		29	x	x		21	x	x	
40	x	x		37	x	x		33	x	x	
50	x	x		42	x	x		41	x		x
65		x	x	50	x	x		48	x	x	x
76	x	x	x	58	x		x	55	x	x	x
84	x	x	x	64	x	x	x	63	x		x
				72	x		x	69	x		x
				79	x		x	77	x		x

### 2.3. Data analysis

Acquired data was analyzed before developing the model in order to expose their characteristics and to see the differences between the three experiments performed. To this end, three different analysis were performed: (i) the disease severity evolution over time, (ii) the mean reflectance versus the wavelengths considered, and (iii) the correlation between the disease severity evolution and the reflectance data. The first analysis observed and justified the variations of the disease severity during the duration of the experiment for the experiments considered. The second analysis studied the reflectance and the FDR signals extracted from leaf reflectance spectra and the canopy reflectance spectra. Finally, the third analysis calculated the Pearson correlation coefficient and the determination coefficient between the disease severity severity and the reflectance or FDR data for each wavelength acquired.

### 2.4. Data processing overview

**Fig 2:**
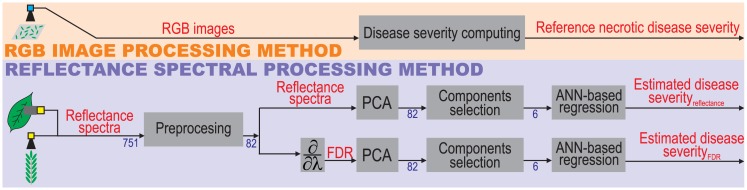
Overview of the processing method performed in this study.

Fig 2 shows an overview of the block diagram of the processing methods carried out in this study. It presents an *RGB image processing* method and a *reflectance spectra processing* method.

The *RGB image processing* method estimates the disease severity by computing the proportion of the total leaf surface covered with necrotic lesions in RGB images of crop leaves. The values obtained were used as a ground truth reference to check the accuracy of the *reflectance spectra processing* method. Although the chlorotic disease is also part of ALS infection, the leaf area with chlorotic disease was not considered into the disease level calculation because leaf chloroses can be associated to other diseases such as plant nutrition problems. For this reason, we considered the necrotic area ratio as the ground truth reference in our study, which is going to be referred henceforth as reference necrotic disease severity.

The *reflectance spectra processing* method estimates the disease severity of the crop as a function of the leaf or canopy spectrum data. This method includes the computation of the FDR spectrum, the conversion to another coordinate space by means of the PCA statistical procedure, the selection of the most relevant components in the new coordinate space, and the regression process implemented by an ANN to estimate the reference necrotic disease severity values.

The *RGB image processing* and the *reflectance spectra processing* methods include supervised learning techniques. Blocks of both methods were implemented in MATLAB^®^.

### 2.5. RGB image processing method

Disease severity values of the crop obtained by the *Disease severity computing* block were taken as the ground truth reference necrotic disease severity. The reference necrotic disease severity was computed from the RGB images of leaves as the proportion of the total leaf surface covered with necrotic lesions [27]. The *Disease severity computing* block obtained the reference necrotic disease severity values of each experiment following the next three steps:

- Step 1: A representative leaf RGB image, which contained parts with disease and parts without disease, was chosen. Selected from this image were (i) 50 pixels from asymptomatic green areas, (ii) 50 pixels from light brown areas with chlorosis, (iii) 50 pixels from dark brown areas with necrosis, and (iv) 50 pixels from the white background.
- Step 2: Pixels of all the RGB images were associated with the asymptomatic class, with the chlorosis class, with the necrosis class, or with the background class, employing a Quadratic Discriminant Analysis classifier [42]. This classifier was trained employing the observations corresponding to each one of 50-pixel groups previously selected to adjust its parameters. It assigned an observation *x* to the class *k* for which
  $$\delta_{k}\left(x\right)=-\frac{1}{2}log|\sum_{k}|-\frac{1}{2}{\left(x-\mu_{k}\right)}^{\prime }\sum_{k}^{-1}\left(x-\mu_{k}\right)+log\left(\pi_{k}\right)$$ (1)
  was the largest, *μ*_*k*_ being the average of the training observations for the *k*^*th*^ class, *π*_*k*_ the proportion of the representative image considered in Step 1 that belongs to the *k*^*th*^ class, and Σ_*k*_ the covariance matrix for the *k*^*th*^ class.
- Step 3: For each RGB image collected in the experiment (Fig 1c), the reference necrotic disease severity was computed as the number of pixels of the areas with necrosis, divided by the sum of pixels of asymptomatic areas, areas with necrosis, and areas with chlorosis.

### 2.6. Reflectance spectra processing method

The spectra processing carried out in this study was performed by the *Preprocessing*, *Derivative*, *PCA*, *Components selection* and *ANN-based regression* blocks, according to the block diagram shown in Fig 2.

#### 2.6.1 Preprocessing block

The *Preprocessing* block performed three actions in each spectrum captured by the spectroradiometer: it applied a *K*_*decimate*_ moving average filter, downsampled the spectrum in a *K*_*decimate*_ to 1 rate, and removed data outside the interest spectral regions. In the research work performed in this work, a decimate rate *K*_*decimate*_ = 5 and the interest regions from blue to NIR (440–850 nm) and from red to NIR (630–850 nm) were considered. It made that the reflectance preprocessed data was composed by 83 and 45 data points for the data from blue to NIR and the data from red to NIR respectively.

#### 2.6.2 Derivative block

The *Derivative* block computed the derivative spectrum from the reflectance spectrum. The derivative value at each wavelength was computed as

$${\frac{ds}{d\lambda }|}_{i}\;=\;\frac{s(\lambda_{i+1})-s(\lambda_{i-1})}{2∙\Delta \lambda }$$ (2)

where *s* represents the reflectance spectrum, *λ* the wavelength, Δ*λ* the wavelength resolution (Δ*λ = λ*_*i*+1_ − *λ*_*i*_), and *i* the wavelength index of both the spectrum and the derivative vectors.

#### 2.6.3 PCA block

The *PCA* block applied a PCA to both the reflectance and the FDR data. PCA is a technique that transforms the input reflectance data to another multidimensional orthogonal coordinate space. PCA defines the first principal component as the one with the highest possible variance, and the rest of the components as the ones with the highest possible variance that are orthogonal to all the previous components. The input signals of the *PCA* block were normalized to calculate the PCA of a set of variables with zero mean and unit variance.

#### 2.6.4 Components selection block

The *Components selection* block selected the components of the PCA performed in the *PCA* block that contained most of the variance, and removed the rest of the components because they contained only a small percentage of the variance of the original data. The number of components selected in this work, which is going to be referred to as *N*_*comp*_, was varied in order to perform a comparison and choose the optimal option.

#### 2.6.5 ANN-based regression block

The *ANN-based regression* block implements a backpropagation MultiLayer Perceptron (MLP) ANN with a single hidden layer. This ANN has *N*_*comp*_*+1* inputs, which are the *N*_*comp*_ components selected by the previous block and the number of Days After Planting (DAP). DAP is the number of days elapsed between the day when the beans were planted and the day when the data was acquired. The hidden layer of the ANN has a variable number of neurons, which is going to be referred to as *N*_*hidden*_, with a *logsig* activation function. In addition, the MLP ANN has one output, which is the estimated disease severity, with a linear activation function. A different ANN was designed for each one of the evaluation scenarios employed in this article to assess the proposed method, using samples from 30% of the plots for testing the ANN and the remaining 70% of the plots for training and validation: 75% of these latter plots were randomly selected to train the ANN and the other 25% to validate it. Moreover, for each evaluation scenario considered in this article different ANNs with the number of hidden neurons between 2 and 25 were evaluated, with 100 iterations for each number of hidden neurons. The ANN tested on each scenario was the one with the lowest estimation error with the training and validation samples.

## 3. Results

An analysis of the data acquired from the study site and an evaluation of the proposed methodology is presented in this section.

### 3.1. Data analysis results

The crop in the experiments grew as expected, being the average productivities 1621, 1725, and 1261 kg/ha for UFV1, UFV2, and FEVP3, respectively. The disease appeared at the flowering stage, and the highest severity was found at the pod maturation stage.

**Fig 3:**
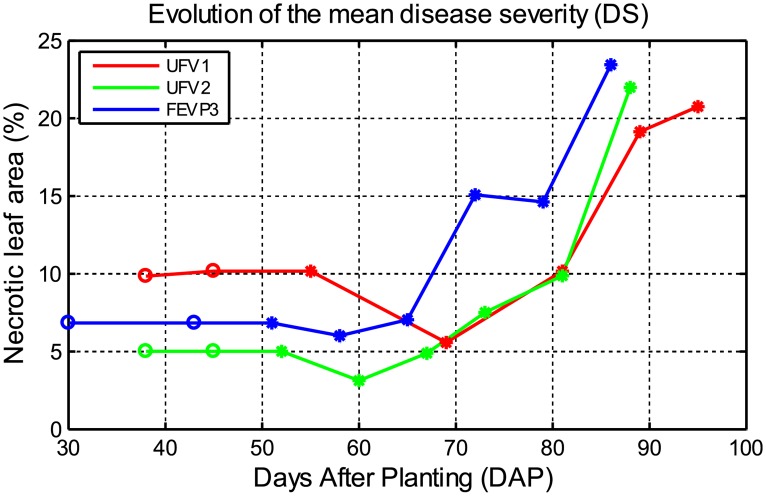
Evolution of the mean reference necrotic disease severity for each one of the three experiments conducted. The reference necrotic disease severity was estimated as the proportion of the total leaf surface covered by necrotic lesions. The decrease of necrotic area between 50 and 80 DAP was caused by the controlled applications of fungicide and the emergence of new leaves.

Fig 3 shows the mean reference necrotic disease severity values for each experiment. The reference necrotic disease severity was estimated from the area of necrosis found in the images of the leaves. As expected, the reference necrotic disease severity generally increased with time, showing some slight decreases between 50 and 80 DAP due to the controlled application of fungicides and the emergence of new leaves. Moreover, the mean values of the reference necrotic disease severity for the different plots of each experiment was 11.79, 7.50, and 9.79 and the standard deviation was 5.73, 3.64, and 3.71 for the experiments UFV1, UFV2, and FEVP3, respectively. It indicates that both the mean value and the variability of the reference necrotic disease severity among the plots of the experiment UFV1 was higher than the variability of the other two experiments.

**Fig 4:**
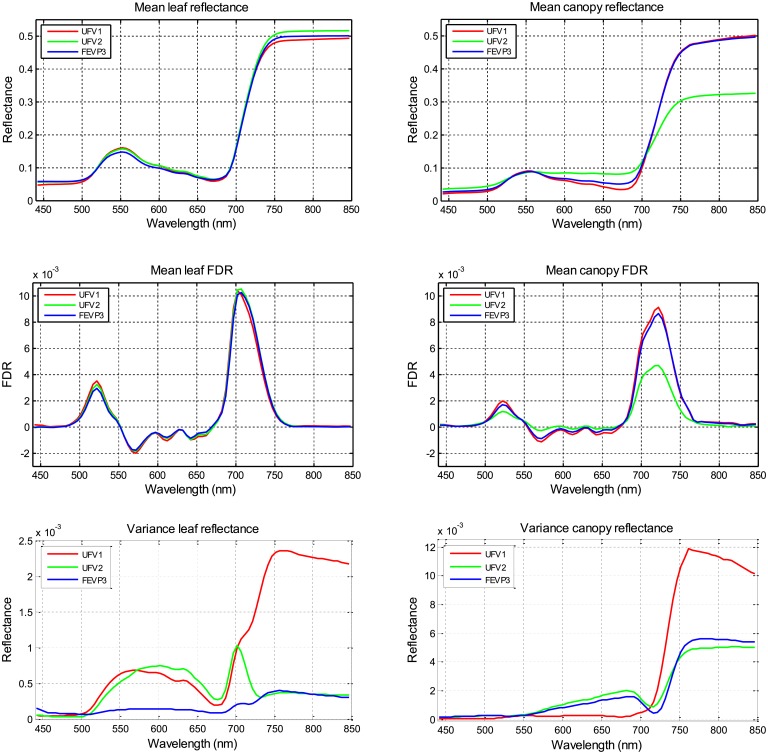
Mean reflectance spectra of (**a**) the leaf and (**b**) the canopy. Mean FDR spectra of (**c**) the leaf and (**d**) the canopy. Variance of (**e**) the leaf and (**f**) the canopy reflectance spectra.

Fig 4 shows, for the leaf and canopy data acquired, the mean reflectance spectra, the mean FDR spectra, and the variance of the raw reflectance spectra in the 450–850 nm waveband. These mean reflectance spectra are in agreement with the ones obtained for bean crops by Blackburn [43]. Fig 4b and 4d show that, in the UFV2 experiment, the canopy reflectance and FDR values for wavelengths greater than 700 nm were slightly lower in comparison to the two other experiments. This resulted from the underdevelopment of the crop, which was caused by a weed infestation.

**Fig 5:**
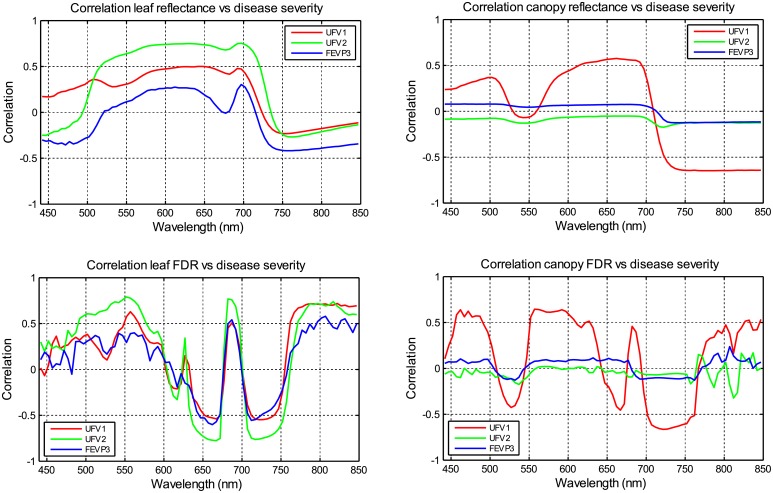
In 440–850 nm, correlation between the reference necrotic disease severity and (a) the leaf reflectance, (b) the canopy reflectance, (c) the leaf FDR, and (d) the canopy FDR. Each graph represents, for each wavelength, the correlation between the reference necrotic disease severity and either the reflectance or the FDR spectra value at this wavelength, considering the different plots and the different acquisition times.

Fig 5 presents the correlation between reference necrotic disease severity measurements and the reflectance spectra or the FDR spectra. These graphs represent, for each wavelength, the correlation between the reference necrotic disease severity and either the reflectance or the FDR spectra value at this wavelength, considering the different plots analyzed in each experiment and the data acquired at different stages of the plant lifecycle for each plot. It is interesting to note the differences between the experiment UFV1 and the other two experiments. Analyzing the data, it may be due to the variability of the reference necrotic disease severity data for this experiment, which causes also that the variance of the leaf and the canopy reflectance for this experiment in the red-NIR spectral region, which is the spectral band where the effects of the disease appears according to the literature [15–17], is higher than the variance of the other two experiments.

### 3.2. Methodology evaluation results

**Fig 6:**
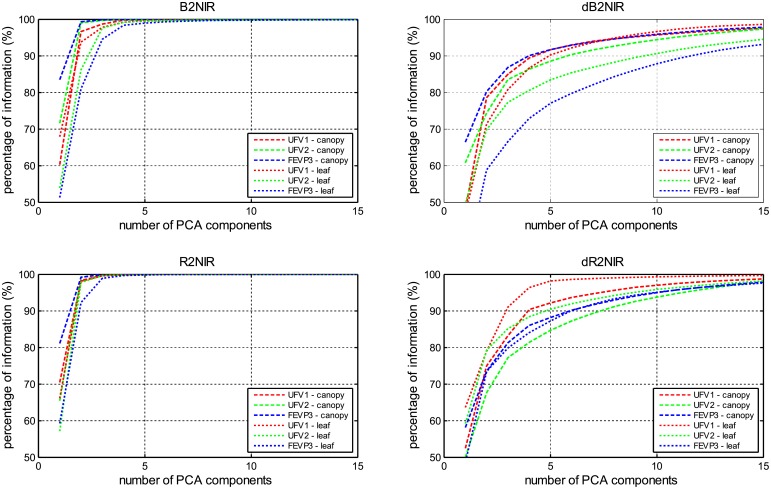
Percentage of information contained in the first components of the PCA of (a) the reflectance data between the blue and the NIR spectral regions (B2NIR), (b) the FDR data between the blue and the NIR regions (dB2NIR), (c) the reflectance data between the red and the NIR regions (R2NIR), and (d) the FDR data between the red and the NIR regions (dR2NIR). B2NIR and dB2NIR signals had 83 data points and R2NIR and dR2NIR signals had 45 data points.

This section presents the results of the tests performed in the different scenarios employed to evaluate the proposed methodology. The first set of evaluation scenarios were employed to analyze the *N*_*comp*_ parameter, i.e., the number of components of the PCA that should be considered in the proposed methodology. To do so, the percentage of information contained in the first components of the PCA was calculated, obtaining the results presented in Fig 6.

The second set of evaluation scenarios were employed to assess the performance of the proposed methodology for the different conditions considered in this article: using leaf reflectance spectral data or canopy reflectance spectral data, using data acquired from the reflectance or the FDR, and using the information from the blue spectral region to the NIR region or from the red region to the NIR region, which contain the wavelengths from 440 to 850 nm and from 630 to 850 nm, respectively. Moreover, these scenarios considered the following values for the *N*_*comp*_ parameter: 3, 5, 6, 8, 10, 12, and 15. Performance of the methodology was evaluated considering the Root Mean Square Error (RMSE) and the determination coefficient, which was obtained as the squared value of the Pearson correlation coefficient. The results obtained in this experiment are presented in Tables 2 and 3.

**Table 2:** RMSE for the evaluation scenarios proposed to assess the methodology, where *N*_*comp*_ refers to the number of significant components chosen from the Principal Component Analysis (PCA); E1, E2, and E3 refer to the experiments UFV1, UFV2, and FEVP3 respectively; L and C refer to the leaf and the canopy spectra respectively; B-NIR and dB-NIR refer respectively to the reflectance and the FDR data between the blue and the NIR spectral regions; and R-NIR and dR-NIR refer respectively to the reflectance and the FDR data between the red and the NIR regions. The wavelength ranges considered in these scenarios were from 440 to 850 nm for the B-NIR and dB-NIR data, and from 630 to 850 nm for the R-NIR and dR-NIR data. The minimum and maximum RMSE values of each column were highlighted with bold and bold-and-italic format respectively.

N_comp_			3	5	6	8	10	12	15
**E1**	**L**	B-NIR	4.49	4.94	4.98	4.75	4.29	4.61	4.23
		dB-NIR	4.46	4.21	4.30	4.05	4.20	4.17	4.47
		R-NIR	4.74	4.65	4.13	4.83	4.22	4.15	4.81
		dR-NIR	4.04	4.20	4.29	4.34	4.13	4.20	4.57
	**C**	B-NIR	7.01	6.86	6.69	6.74	***6*.*88***	6.93	7.05
		dB-NIR	6.87	6.93	6.56	6.94	6.85	6.84	6.87
		R-NIR	6.81	7.03	6.72	6.80	6.73	***6*.*97***	***7*.*37***
		dR-NIR	***7*.*11***	***7*.*06***	***6*.*92***	***7*.*05***	6.87	6.93	7.01
**E2**	**L**	B-NIR	3.88	3.70	3.16	3.52	3.64	3.65	3.48
		dB-NIR	3.08	3.54	3.57	3.44	3.53	3.48	3.81
		R-NIR	4.36	3.60	3.72	3.79	4.01	4.50	4.10
		dR-NIR	3.05	3.36	3.64	3.64	3.51	3.82	3.76
	**C**	B-NIR	2.73	2.46	3.08	2.40	2.82	2.89	2.71
		dB-NIR	2.63	2.65	2.68	2.73	2.76	2.47	2.95
		R-NIR	2.43	2.71	2.69	2.58	2.69	2.59	2.84
		dR-NIR	2.76	2.60	2.63	2.67	2.97	2.64	2.79
**E3**	**L**	B-NIR	3.72	3.75	3.79	3.67	3.39	4.09	3.35
		dB-NIR	4.19	3.61	3.56	3.65	3.89	4.00	3.34
		R-NIR	4.09	3.82	3.77	4.09	3.85	3.98	3.73
		dR-NIR	3.61	3.68	3.85	3.63	3.92	3.96	3.21
	**C**	B-NIR	**2.43**	2.42	2.43	2.32	2.44	**2.21**	2.43
		dB-NIR	2.43	2.45	**2.37**	2.42	**2.16**	2.42	2.37
		R-NIR	2.43	**2.39**	2.41	2.47	2.43	2.25	2.45
		dR-NIR	2.48	2.47	2.41	**2.27**	2.28	2.51	**2.31**

**Table 3:** Determination coefficient for the evaluation scenarios proposed to assess the methodology, where *N*_*comp*_ refers to the number of significant components chosen from the Principal Component Analysis (PCA); E1, E2, and E3 refer to the experiments UFV1, UFV2, and FEVP3 respectively; L and C refer to the leaf and the canopy spectra respectively; B-NIR and dB-NIR refer respectively to the reflectance and the FDR data between the blue and the NIR spectral regions; and R-NIR and dR-NIR refer respectively to the reflectance and the FDR data between the red and the NIR regions. The wavelength ranges considered in these scenarios were from 440 to 850 nm for the B-NIR and dB-NIR data, and from 630 to 850 nm for the R-NIR and dR-NIR data. The maximum and minimum determination coefficient values of each column were highlighted with bold and bold-and-italic format respectively.

N_comp_			3	5	6	8	10	12	15
**E1**	**L**	B-NIR	0.62	0.58	0.54	0.58	0.65	0.62	0.67
		dB-NIR	0.64	0.66	0.65	0.69	0.68	0.67	0.68
		R-NIR	0.58	0.61	0.69	0.60	0.67	0.68	0.58
		dR-NIR	0.69	0.68	0.66	0.65	0.68	0.67	0.62
	**C**	B-NIR	0.56	0.55	0.55	0.54	0.54	0.54	0.55
		dB-NIR	0.54	0.54	0.59	0.58	0.57	0.56	0.55
		R-NIR	0.56	0.55	0.56	0.54	0.55	0.55	0.49
		dR-NIR	0.53	0.52	0.55	0.52	0.57	0.55	0.53
**E2**	**L**	B-NIR	0.81	0.81	**0.86**	0.83	0.81	0.83	**0.83**
		dB-NIR	**0.87**	0.85	0.82	**0.84**	0.82	**0.83**	0.79
		R-NIR	0.71	**0.85**	0.81	0.79	0.80	0.76	0.78
		dR-NIR	0.86	0.85	0.84	0.82	**0.83**	0.82	0.81
	**C**	B-NIR	0.15	0.24	0.11	0.28	0.17	0.26	0.19
		dB-NIR	0.18	0.09	0.16	0.09	0.33	0.21	0.29
		R-NIR	0.22	0.14	0.13	0.18	0.17	0.24	0.25
		dR-NIR	0.12	0.15	0.18	0.16	0.10	0.13	0.13
**E3**	**L**	B-NIR	0.69	0.70	0.68	0.67	0.72	0.66	0.75
		dB-NIR	0.65	0.71	0.71	0.72	0.65	0.62	0.73
		R-NIR	0.64	0.68	0.67	0.64	0.65	0.63	0.70
		dR-NIR	0.70	0.69	0.70	0.71	0.64	0.67	0.77
	**C**	B-NIR	0.13	0.10	***0*.*09***	0.13	0.08	0.28	0.12
		dB-NIR	0.09	0.07	0.14	0.15	0.27	0.11	***0*.*10***
		R-NIR	0.08	0.08	0.12	***0*.*08***	***0*.*08***	0.19	0.14
		dR-NIR	***0*.*05***	***0*.*06***	0.11	0.22	0.17	***0*.*08***	0.15

## 4. Discussion

The different evaluation scenarios assessed in this study made possible to analyze the influence of different parameters within the proposed methodology on estimating the ALS severity in common bean crops. The parameters analyzed were (i) the way the reflectance data is acquired (leaf or canopy), (ii) the variable considered in the analysis (reflectance or FDR data), (iii) the wavelength spectral region that is more relevant for the analysis (from blue to NIR or from red to NIR), and (iv) the number of inputs for the ANN-based regression block, which is related to *N*_*comp*_, the number of components considered as relevant information.

The first observation about the results obtained is that the reflectance measured from the canopy does not achieve results accurate enough to consider it as a possible measurement to estimate the reference necrotic disease severity. It can be calculated from the information of Table 3 that the mean determination coefficient values for all the experiments is 0.71 for the evaluation scenarios with data measured from the leaf, and 0.28 for the evaluation scenarios with data measured from the canopy. Although the leaf area index was not measured during the experiment, the variation on this index during the plant development could explain the lower determination coefficients of canopy measurements than those of leaf measurements. Thus, the variance within the canopy measurements may be mostly related to the growth of the plants. Nevertheless, it is also important to note that the proposed method obtained significant correlations when employed canopy reflectance data, which means that it is possible to detect spectral differences between asymptomatic and infected plants. This was possible because the proposed ANN was trained with reflectance data acquired at different times along the plant development, so it was able to estimate the disease severity index despite of the differences in the leaf area index. For this reason, authors consider that considering the leaf area index or analyzing the differences between the canopy reflectance of the plant and the canopy reflectance of the soil should be considered in further experiments to improve the obtained results.

The second observation is that there are not significant differences between the results of the methods which employed the reflectance signal and those which employed the FDR signal. From the data shown on Tables 2 and 3, it can be seen that neither the reflectance nor the FDR leads to more accurate results compared to the other. Analyzing the mean determination coefficient values, scenarios considering reflectance data obtained a mean determination coefficient of 0.49 and scenarios considering FDR data obtained a mean determination coefficient of 0.50, Analyzing these two groups of data with the two-sample Student’s T-test it can be said that there are not significant differences between the mean values of these two groups with a p-value of 0.78. There are studies in the literature in which the use of the FDR presents advantages [29, 44, 45]. However, other studies concluded that the use of the FDR presents advantages only in specific situations [46, 47], and there are also a few studies in the literature in which the use of the FDR does not present advantages at all [48]. Our study shows neither advantages nor disadvantages by using the reflectance data and the FDR data, which concurs with the literature.

The third observation is that the methods that employed the information of the wavelength spectral region between blue and NIR obtained similar results to the methods that employed the information of the wavelength region between red and NIR. This observation is based on the information presented in Table 3, where it can be calculated that the mean determination coefficients are 0.51 and 0.49 for the scenarios that considered the wavelength region from blue to NIR and from red to NIR, respectively. It can be explained because the spectral behavior of the bean plants in the visible spectral region (400–700 nm) depends on the content of plant pigments. Chlorophyll, which is a pigment considered as a very important agronomic parameter to evaluate the growth and health of the plant presented two absorption peaks in the blue and the red regions while others pigments present absorption peak just in the blue region [49]. Thus, several authors of the literature considered the red spectral region as the most important region because we could isolate the chlorophyll content estimation with this region. The presented results show that there is not any improvement when considering the blue and green spectral regions as input information, which agrees with the theory that considers the red and the NIR regions as the most important to evaluate the plant growing process.

The fourth observation is that most of the variance of the reflectance and FDR signals considered can be comprised with the first components of the PCA, as it can be seen in Fig 6. This observation suggests that most of the last components of the PCA, which are the ones with the least variance, could be discarded in order to minimize the complexity of the proposed method. Moreover, the results presented in Tables 2 and 3 support this observation because there is not a dependence between *N*_*comp*_ and the RMSE or the determination coefficient: for values of *N*_*comp*_ greater than 10 the results obtained do not improve significantly.

Plant disease severity estimation methods can be classified into three types [50]. Type I: visual rating methods, done by raters with the aid of scales or keys or by disease measurement devices. These methods require trained raters that are expensive and suffer the subjectivity of raters, which can be prone to illusions [51]. Type II: image photography and image analysis methods performed in the visible spectrum. These methods employ only the red, green and blue spectral regions of the visible spectra [52]. Type III: hyperspectral imaging methods, which employ images composed by many spectral regions, not just the red, green and blue regions of the visible spectra, and then they have more potential to better estimate plant disease severities [53]. The method of this study belongs to Type III methods.

The results obtained suggest that a method which uses the leaf reflectance for a wavelength region between the red and the NIR wavelengths (630–850 nm) could be employed to estimate ALS severity of common beans crops. Results presented in Tables 2 and 3 suggest this assertion, because the RMSE and the determination coefficient obtained for the methods with the best performance improve the results obtained by other authors of the literature, such as Gnyp *et al [48].* Nevertheless, these results has been obtained for regions of Minas Gerais (Brazil), so extrapolations of the proposed method to other agronomic regions should be validated first. As the reflectance and the FDR obtained similar results, the reflectance was chosen for two reasons: the first is that it requires less processing, as FDR is obtained from the reflectance signal, and the second is that the reflectance signal can include more reflectance variance than the FDR in the first components of the PCA, as can be seen in Fig 6. Moreover, using the wavelength spectral region between red and NIR (630–850 nm), instead of the region between blue and NIR (440–850 nm), requires a simpler reflectance sensor, which can reduce its price, and allow us to work with a smaller region (220 nm vs 410 nm), which reduces the required computational load.

The ground truth used in our work was based on RGB analysis (visible spectral region), which is mainly influenced by the chlorophyll degradation. Thus, someone could think that a sensor using just the visible bands could be efficient to detect ALS disease. However, a commercial sensor should keep the NIR spectral band in order to compensate the physical differences among the leaves since the leaf spectral signatures on NIR region are mainly influenced by the physical characteristics of the leaf.

## 5. Conclusions

In the estimation of ALS disease severity in bean crops by hyperspectral reflectance spectrometry, this study suggests that: (i) successful estimations with coefficients of determination up to 0.87 can be achieved if the spectra are acquired with the spectroradiometer in contact with the leaves; (ii) unsuccessful estimations are obtained when the spectra are acquired by the spectroradiometer from farther than one meter above the crop; (iii) the red to NIR spectral region (630–850 nm) offers the same estimation precision as the blue to NIR region (440–850 nm); and (iv) neither significant improvements, nor significant detriments, are achieved when the input data to the estimation processing system is obtained from the FDR spectra, instead of the reflectance spectra.

## Supporting information

S1 Text Description of the Repository File. Description of the folder’s structure tree of the RAR Repository File of this article. The Repository File of this article can be downloaded from the other 13 compresed files: S1, S2, S3, S4, S5, S6, S7, S8, S9, S10, S11, S12 and S13 Files.
(PDF)

S1 File Part 1/13 of the Repository File. (RAR)

S2 File Part 2/13 of the Repository File. (RAR)

S3 File Part 3/13 of the Repository File. (RAR)

S4 File Part 4/13 of the Repository File. (RAR)

S5 File Part 5/13 of the Repository File. (RAR)

S6 File Part 6/13 of the Repository File. (RAR)

S7 File Part 7/13 of the Repository File. (RAR)

S8 File Part 8/13 of the Repository File. (RAR)

S9 File Part 9/13 of the Repository File. (RAR)

S10 File Part 10/13 of the Repository File. (RAR)

S11 File Part 11/13 of the Repository File. (RAR)

S12 File Part 12/13 of the Repository File. (RAR)

S13 File Part 13/13 of the Repository File. (RAR)
