# Supplementary material for: Leaf and canopy reflectance spectrometry applied to the estimation of angular leaf spot disease severity of common bean crops
Source: PLoS One. 2018 Apr 26;13(4):e0196072. doi: 10.1371/journal.pone.0196072 (PMC5919580; doi:10.1371/journal.pone.0196072)
Supplement: S1 Text — Description of the folder’s structure tree of the RAR Repository File of this article. The Repository File of this article can be downloaded from the other 13 compresed files: S1, S2, S3, S4, S5, S6, S7, S8, S9, S10, S11, S12 and S13 Files. (PDF) [file pone.0196072.s001.pdf]

The folders' tree structure of this repository is presented in as Figure 1.

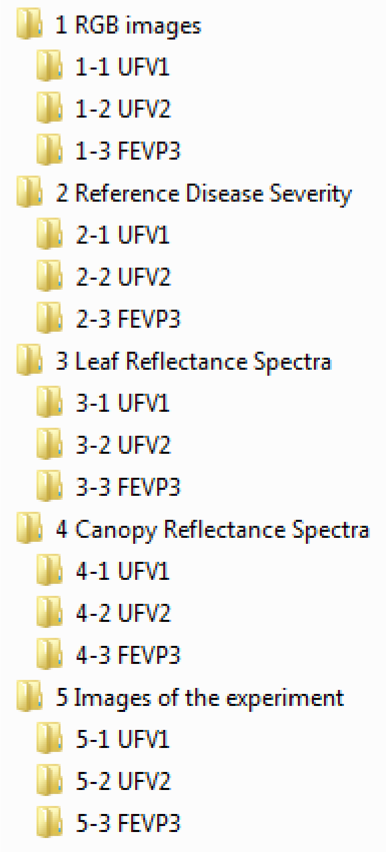

Figure 1: Tree structure of the respository

There are 5 main directories: "**1 RGB images**", "**2 Reference Disease Severity**", "**3 Leaf Reflectance Spectra**", "**4 Canopy Reflectance Spectra**", and "**5 Images of the experiment**". The first four directories contain the data associated to the different input and output information in Figure 2. The last directory contains illustrative images of the experiments performed.

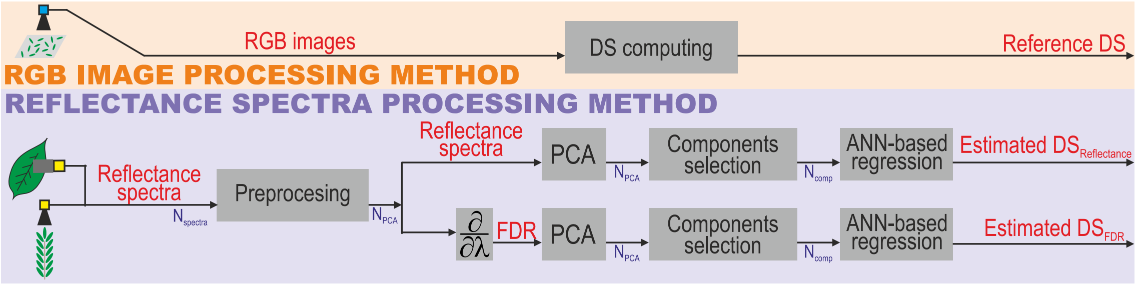

Figure 2: Overview of the processing method performed in this article, where some of the data contained in the repository is shown: RGB images, Reference Disease Severity (DS), and (canopy and leaf) Reflectance spectra.

Each one of the five previously mentioned directories contains 3 subfolders, related with each one of the three experiments performed: UFV1, UFV2 (both of them performed in Viçosa, MG, Brazil), and FEVP3 (performed in Oratorios, MG, Brazil).

The content of each folder is explained in the next sections.

Archives in **1 RGB images** folder

This folder includes images in “jpg” format of a set of leaves of the crop laced at the bottom of a wooden box. Figure 3 presents, as example, one of these photos.

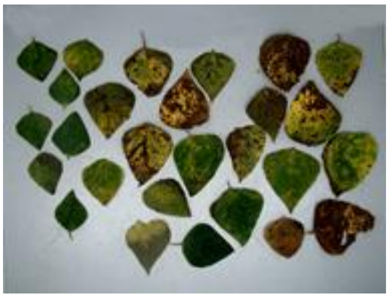

Figure 3: An example of RGB images taken of a set of leaves, which were placed inside of a wooden box.

Archives in **2 Reference Disease Severity** folder

This directory includes Comma Separated Value (CSV) files with “.csv” extension obtained after applying the “RGB image processing method” shown in Figure 2. Each row of these files contains the information of a parcel, having the parcel number in the first column and the disease severity value in the second column. The names of these files specifies the name of the experiment in the first letters and the acquisition date in the last numbers, where the date format was *yyyymmdd* where *yyyy* refers to the year, *mm* to the month, and *dd* to the day.

Archives in **3 Leaf Reflectance Spectra** folder

This directory contains CSV files with “.txt” extension that the HandHeld-2 spectroradiometer captured. Each row of the file contains the wavelength (in nm) in the first column and the reflectance value in the second column. The first letters of the file identify the experiment. The following four numbers correspond to the parcel. The final numbers correspond to repetitions of the same capture. Figure 4 presents a photo of the Leaf Reflectance Spectra acquisition procedure.

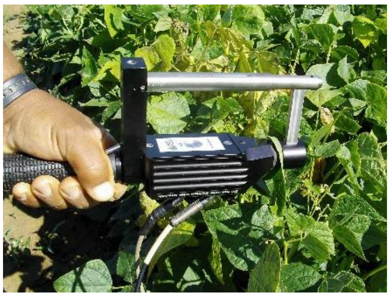

Figure 4: Photo of the Leaf Reflectance Spectra acquisition procedure.

Archives in **4 Canopy Reflectance Spectra** folder

This folder contains CSV files with ".txt" extension that the HandHeld-2 spectroradiometer captured. Each row of the file contains the wavelength (in nm) in the first column and the reflectance value in the second column. The first letters of the file identify the experiment. The following four numbers correspond to the parcel. The final numbers correspond to repetitions of the same capture. Figure 5 presents a photo of the Canopy Reflectance Spectra capturing process using a platform with wheels.

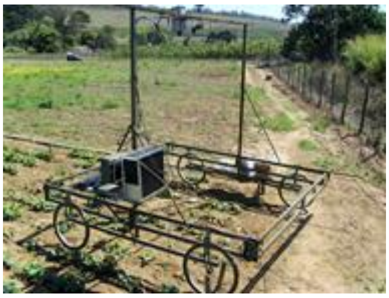

Figure 5: Photo of the Canopy Reflectance Spectra acquisition process using a platform with wheels.

Archives in **5 Images of the experiment** folder

This folder of the repository includes some representative photos of the experimental areas, as for example, the shown in Figure 6.

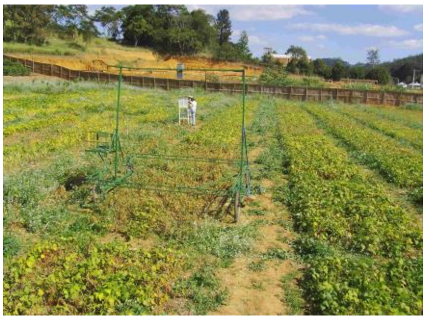

Figure 6: An example image of the experiment UFV1, performed in Viçosa, MG, Brazil.
